# Translation of Pharmacodynamic Biomarkers of Antibiotic Efficacy in Specific Populations to Optimize Doses

**DOI:** 10.3390/antibiotics10111368

**Published:** 2021-11-09

**Authors:** Manjunath P. Pai, Ryan L. Crass

**Affiliations:** 1 Department of Clinical Pharmacy, College of Pharmacy, University of Michigan, Rm 2568, 428 Church St., Ann Arbor, MI 48109, USA; 2 Ann Arbor Pharmacometrics Group, Ann Arbor, MI 48108, USA; Ryan.Crass@a2pg.com

**Keywords:** special populations, pharmacokinetics, antimicrobials, exposure–response, modeling, simulation

## Abstract

Antibiotic efficacy determination in clinical trials often relies on non-inferiority designs because they afford smaller study sample sizes. These efficacy studies tend to exclude patients within specific populations or include too few patients to discern potential differences in their clinical outcomes. As a result, dosing guidance in patients with abnormal liver and kidney function, age across the lifespan, and other specific populations relies on drug exposure-matching. The underlying assumption for exposure-matching is that the disease course and the response to the antibiotic are similar in patients with and without the specific condition. While this may not be the case, clinical efficacy studies are underpowered to ensure this is true. The current paper provides an integrative review of the current approach to dose selection in specific populations. We review existing clinical trial endpoints that could be measured on a more continuous rather than a discrete scale to better inform exposure–response relationships. The inclusion of newer systemic biomarkers of efficacy can help overcome the current limitations. We use a modeling and simulation exercise to illustrate how an efficacy biomarker can inform dose selection better. Studies that inform response-matching rather than exposure-matching only are needed to improve dose selection in specific populations.

## 1. Introduction

Antimicrobial drug development faces numerous challenges that extend beyond fundamental limitations in discovery platforms [1]. Potential lead compounds advance through multiple hurdles to reach the final stage of development, which is to establish their efficacy often based on a fixed or weight-based dose that may be adjusted for kidney function. The current approach relies on two well-orchestrated phase 3 clinical trials to make this efficacy determination. Phase 3 clinical trials performed in patients with infectious diseases, especially antibacterial agents, include hundreds of patients and employ non-inferiority designs. For instance, a recently published phase 3 study randomized 145 patients to the intervention arm (cefiderocol) and 147 patients to the comparator (meropenem) [2]. At face value, these numbers are an order of magnitude lower than those of agents approved for other indications, such as cardiovascular disease [3]. However, the execution of this study required the participation of 76 centers in 17 countries, and took several years to complete [2]. This case exemplifies the high cost and complexity of advancing novel antibiotics to the marketplace. Alternate models for regulatory approval include creating a case for lower quantities of clinical efficacy data to support this unmet medical need [4,5]. These range from the acceptance of preclinical data to combinations of a single phase 3 study that is coupled or uncoupled to multiple smaller, pathogen-focused clinical studies [6].

Many questions remain to be answered to ease the tension between staying with the current paradigm or migrating to a new antibiotic drug development approach. A key question that raises debate is, “How confident are we that the information gained from 500 (as an example) patients in a clinical trial environment justifies use in millions of patients?” We currently make the assumption that the efficacy established in two phase 3 clinical trials is sufficient justification [7]. Reliance on less stringent clinical trial data would therefore be perceived as an approach that could lower this confidence. Regulators are acutely aware of this problem and have launched programs to bridge this translational gap [8]. Model-informed drug development is one such approach that works to build exposure–response relationships that integrate data from preclinical and clinical sources [8]. Antimicrobial drug development in particular has benefited from this approach, by translating pharmacokinetic–pharmacodynamic relationships established in vitro and/or in animal models to clinical studies. As with any model, validation is necessary but challenging to perform across the entire end-use clinical population. As noted above, the small total clinical trial sample size leads to an even smaller proportion of individuals within specific populations. Specific populations recognized by regulatory agencies when defining antimicrobial labels include age (pediatric, geriatric), pregnancy and lactation, gender, renal impairment, and hepatic impairment [9]. At present, the acceptance of the drug dosing strategy in these specific populations relies on the assumption that exposure-matching will lead to comparable outcomes. For example, achieving a comparable systemic area under the curve (AUC) in elderly patients to those that are young should lead to comparable outcomes. Likewise, ensuring comparable AUC/MIC values accounts for the pathogen and not potential differences in host response. However, we have multiple case examples where achieving similar systemic exposure values in specific populations may not necessarily lead to comparable clinical outcomes [10]. Patients with impaired kidney function have been shown to have worse clinical outcomes than patients without impairment that cannot currently be explained by exposure alone [11,12,13]. Likewise, the clinical outcomes in patients with certain comorbid conditions such as diabetes may be worse than patients without diabetes despite having comparable exposures [14]. The current review outlines the current framework, limitations, and opportunities to improve dosing in specific populations. We explicitly compare the potential of response-matching using conventional and alternate biomarkers of efficacy that can support dose optimization beyond exposure-matching. The use of the term biomarker in this review is a characteristic that is objectively measured as an indicator of pharmacologic response to a therapeutic intervention.

## 2. Exposure-Matching as a Surrogate for Efficacy

The practice of extrapolating efficacy findings from the primary evaluated adult population to specific populations such as pediatric patients has a long track record of use [10]. The drive for use of this approach has been to some extent the Best Pharmaceuticals for Children Act (BPCA), a law enacted in 2002 [15]. This law grants a 6-month extension to patent exclusivity when product labels carry an indication with dosing information for children. Multiple barriers including the availability of pediatric formulations, limited sample size, lack of efficacy data, and others have hindered the goals of the BPCA. On the other hand, the use of exposure-matching has helped to improve pediatric use labeling, though much work is needed to fully address this lag in labeling [16,17]. When the disease course and the response to the antibiotic are expected to be similar in adult and pediatric populations, then pharmacokinetic (PK) and safety data from adults can be used as a benchmark for dosing in children. So, in this case, we must (and often do) assume that similar exposures lead to similar clinical outcomes in adults and children. However, a uniform definition of “similar” has not been established and surveys of antimicrobial product submissions have shown variability in criteria for exposure-matching [18]. In most cases, the AUC from time zero to infinity (AUC_0-inf_) or AUC from time zero to the last measurable concentration (AUC_0-t_) have been used as the relevant exposure metric. Some key examples for justifying similarity have included: (A) median or mean pediatric AUC within 20% of the reported adult value; (B) median or mean pediatric AUC within 50% of the reported adult value; (C) ≥75% of the pediatric AUC range within the adult AUC range; (D) geometric mean ratio of the pediatric to adult AUC between 0.8 and 1.2 [18].

Similar to the above case for translation across the life span, comparable problems exist with optimal dose selection in patients with organ dysfunction. Patients with abnormal kidney and liver function are often excluded from clinical trials [19]. Dedicated PK studies in these specific populations also do not even have to occur in series. That is, a dedicated PK study in subjects (not infected) with abnormal kidney function is sometimes performed after or during the phase 3 clinical trials; therefore, these data may or may not inform dosing for the registrational studies. Alternatively, population PK models may be developed using pooled data from across the clinical program and used to inform labeling in renal impairment without direct evaluation in the phase 3 clinical trial [20]. Again, the underlying assumption is that the disease course and the response to the antibiotic are expected to be similar in patients with and without organ dysfunction. To most practicing clinicians, this assumption is likely unacceptable. Unfortunately, the exclusion of these populations due to safety concerns and ethical and trial efficiency reasons leads to a lack of data to test this assumption. Another conundrum includes the choice of the reference group for exposure. Often, models are built with the selection of normal kidney function (e.g., glomerular filtration rate >90 mL/min) as the reference group. However, the underlying distribution of the phase 3 clinical trial population (especially in infectious diseases) may be centered around the mild to moderate impairment group [21]. This approach may lead to unnecessary dose reductions with exposure-matching and a lack of considerations for increasing doses in patients with augmented kidney function. An exception to this rule includes the dosing recommendations for cefiderocol, which include an additional dose per day in patients with creatinine clearance ≥120 mL/min [22]. The recognition of this potential dose modification by regulators opens the door for further consideration of this principle. However, as in the pediatric case scenario presented above, similarity in exposure can be established based on a match to point estimate, confidence interval of mean effect, or a range of exposures observed in clinical trials [10,21]. Ultimately, guaranteeing exposure similarity cannot consistently guarantee exposure–response similarity across specific populations without explicit evaluation of this assumption. Modeling the exposure–response relationships for relevant clinical trial endpoints and biomarkers of efficacy is necessary to improve upon the current paradigm.

## 3. Clinical Trial Endpoints for Exposure–Response Analyses

Market entry for new antibiotics in the past two decades has followed a familiar pathway of gaining approval for acute bacterial skin and skin structure infections (ABSSSI), complicated urinary tract infections (cUTI), and community-acquired pneumonia (CAP) as an initial indication. Approval for more difficult to treat nosocomial pneumonia indications, including hospital-acquired bacterial pneumonia (HABP) and ventilator-associated bacterial pneumonia (VABP), has been subsequently sought thereafter. These therapeutic indications have well defined regulatory guidance that informs disease definition, enrollment criteria, efficacy endpoints, and statistical and labeling considerations [23,24,25,26]. In contrast, approval for indications such as bloodstream infections, endocarditis, meningitis, and osteomyelitis are rarely sought due to cost, complexity, and regulatory uncertainty. Table 1 includes a summary of current regulatory guidance on primary and secondary endpoints as measures of efficacy. As shown, most endpoints are discrete rather than continuous, such as all-cause mortality at a specified time point or microbiological eradication at the site of infection. The data captured for these discrete endpoints allow for time-to-event (survival) analyses to quantify efficacy [27]. Ordered categorical data captured through scoring systems such as the clinical pulmonary infection score could also in theory be modeled as the response variable. These scoring systems include continuous variables such as temperature, white blood cell counts, and oxygenation coupled with a semi-quantitative measure of tracheal secretions and bacterial culture profiles [28]. Exposure–response models can be developed for all endpoints, including discrete and ordered categorical data; however, these models have a limited ability to inform adaptive dosing interventions in clinical practice based on a quantifiable, real-time measure of response. For example, let us assume an exposure–response model is constructed for ABSSSI to predict the probability of achieving a ≥20% reduction in lesion size by 72 h. A clinician observes a 10% reduction in lesion size at 20 h and a 13% reduction in lesion size at 48 h. Should the dose be increased or maintained to ensure an adequate clinical response by 72 h? This question cannot be easily answered when the endpoint is discrete. Conversely, models linking continuous individual exposure to continuous response endpoints (i.e., biomarkers) facilitate the assessment of the impact of patient- and disease-related factors on both exposure and response. Covariates that shift this exposure–response relationship can then help us discern whether dose modification may or may not be needed in specific populations, and individual patient monitoring can include dose adaptation based on both measures of exposure (e.g., drug concentrations) and response (e.g., biomarker levels) in the setting of a narrow therapeutic index.

Numerous well-designed registration studies have been performed with antibiotics to treat ABSSSI since the publication of industry guidance [23,29,30,31,32,33,34,35]. As noted in Table 1, the quantitative metric of percent reduction in lesion size within 48–72 h is used as the primary endpoint. Refinement of this outcome measure is specifically addressed in current guidance by moving beyond the multiplication of length and width for surface area [23]. Imaging technologies exist to translate photos of the lesion and convert them to surface area measurements that in theory could be performed on a daily or twice daily basis to capture time-course data [36]. Building an exposure–response relationship on this regulatory accepted clinical endpoint would in theory allow for improved granularity of differences in outcomes in specific populations. Likewise, modeling exposure to changes in quantitative urine culture data could also inform exposure–response relationships among specific populations with cUTI [37]. These approaches could also be coupled with systemic biomarkers of inflammation and infection to better characterize pharmacologic effects.

## 4. Systemic Efficacy Biomarkers of Inflammation and Infection

Clinical endpoints serve as useful end-of-treatment response metrics but as noted above are discrete values that limit the construction of useful exposure–response models to aid extrapolation. Alternatives, such as the direct quantification of bacterial load within tissues that are infected, are also not always feasible or reliable when ascertaining the clinical pharmacologic efficacy of antibiotics. We recognize that host response mechanisms play a pivotal role in the outcome of infectious diseases that impact morbidity, mortality, length of stay, and therapy duration. Patients with neutropenia, for example, have far worse outcomes than patients who have normal neutrophil counts, and one would expect the need for higher antibiotic exposures in the former compared to the latter population to achieve comparable effects [38]. Changes in immune response biomarkers therefore have the potential to serve as useful surrogate biomarkers of efficacy across specific populations. Four key biomarkers of efficacy have been studied across multiple infectious disease conditions to serve in this role, and include: (1) procalcitonin; (2) C-reactive protein (CRP); (3) Interleukin-6; and (4) presepsin [39,40,41,42].

While several new biomarkers have been identified, none have matured sufficiently for clinical use, with the exception of procalcitonin [43]. Procalcitonin production is induced in thyroid cells during bacterial infections and tissue injury [43]. Viral infections induce the production of interferon-gamma, which suppresses the production of procalcitonin [43]. This distinction can help guide antibiotic treatment especially for respiratory tract infections that can have multiple etiologies. Recently, the rate of reduction in procalcitonin concentrations has been used to guide antibiotic treatment duration, and so can serve as a useful surrogate of efficacy [44]. The concentration–time profile of biomarkers such as procalcitonin has been characterized in health volunteers challenged with lipopolysaccharides (LPS), as well as in patients with abnormal kidney function [45,46]. These prior studies indicate that procalcitonin concentrations rise within 3–4 h after an LPS challenge and achieve a peak concentration within 6 to 24 h [45,47]. The expected half-life of procalcitonin is 24 h, and so changes in the dynamics of this profile can be correlated to treatment effects. For example, faster clearance of procalcitonin would imply a better response to therapy than slow clearance in a non-responder. The complexity of modeling endogenous substrates, however, requires a good understanding of the kinetics of production and degradation. A recent analysis based on daily measurements of procalcitonin in patients with sepsis has allowed for the construction of such a model that includes initial conditions at the start of therapy, a lag time for response to therapy, first-order production and degradation rate constants for procalcitonin, and random effect terms to account for treatment variation and immune response [48]. These non-linear mixed effects models extend the simple single-point interpretations of biomarkers such as procalcitonin by integrating repeated measures and forecasting the response to therapy. The extension of these approaches to other biomarkers could further aid translation across specific populations and different infectious disease indications.

## 5. Case Study Illustrating Exposure–Response-Matching Using Biomarkers

Clinical trial simulations of vancomycin treatment and procalcitonin response were performed to illustrate the potential value of exposure–response modeling as a means to improve the precision of antibiotic therapy in specific populations. Simulations were performed in NONMEM (Version 7.4) and processed using R (Version 3.6.3).

Each of the 100 simulated trials consisted of a unique sample of 125 adult patients to approximate the sample size of a major randomized clinical trial of treatment for *Staphylococcus aureus* bacteremia and endocarditis [49]. Subject covariate vectors (sex, age, weight, body mass index (BMI), serum creatinine, and creatinine clearance (CrCl)) were randomly sampled from the National Health and Nutrition Examination Survey (NHANES) pre-pandemic 2017–2020 datasets [50]. A summary of the covariates in the NHANES datasets is provided in the Supplementary Materials (Table S1).

Vancomycin dosing was 15 mg/kg of total body weight (rounded to the nearest 250 mg) with the dosing interval determined by renal function following a modified Matzke nomogram (Table S2) for a total of 28 days. Individual vancomycin PK parameters and concentration–time profiles were predicted using a previously published two-compartment model with covariate effects of weight on central distribution volume and CrCl on systemic clearance and inter-individual variability on all clearance and volume parameters [51]. The clinical trial simulations did not include adaptive feedback consistent with clinical TDM.

Procalcitonin (PCT) response was predicted using an indirect response model with drug effect parameterized as inhibiting the zero-order production of procalcitonin (*k_in_*) [52]:

$$\frac{dPCT}{dt}=k_{in}\cdot \left(1-\frac{I_{max}\cdot C_{t}}{IC_{50}+C_{t}}\right)-k_{out}\cdot PCT$$

Drug effect was parameterized using a classical $I_{max}$ inhibitory function with the $I_{max}$ set to the theoretical upper limit of 1 (100% inhibition of procalcitonin production) and the vancomycin concentration ($C_{t}$) producing 50% of the maximal response ($IC_{50}$) set to 10 mg/L, which approximates a clinically relevant vancomycin trough concentration. The first-order elimination rate constant of procalcitonin (*k_out_*) was set to 0.0289 h^−1^ (*t_1/2_* 24 h) [48]. The population baseline procalcitonin level was set to 3.6 ng/mL to be representative of a *S. aureus* bacteremia population [53]. At baseline, the procalcitonin level is at steady-state ($\frac{dPCT}{dt}=0$) and vancomycin concentration is zero, such that $k_{in}$ is equal to $k_{out}\cdot PCT_{baseline}$. Inter-individual variability of 33 and 50 percent coefficient of variation (%CV) was included on $PCT_{baseline}$ and $IC_{50}$, respectively.

Two sets of clinical trial simulations were performed. In the first simulation, no covariate effects were included on procalcitonin response parameters. This is the base assumption that the exposure–response relationship is constant across all patient sub-populations. This reflects the current paradigm for precision medicine in specific populations where exposure-matching is equivalent to response-matching. In the second set of simulations, covariate effects BMI and CrCl were included on the $IC_{50}$ for procalcitonin response. This represents the condition in which intrinsic patient factors may influence response independent of exposure where exposure-matching may not equate to response-matching. The covariate effects were described using power functions with a positive value for BMI (higher BMI, higher $IC_{50}$, worse response) and negative value for CrCl (higher CrCL, lower $IC_{50}$, better response). In each of the 100 simulated trials, individual patient vancomycin exposure metrics and procalcitonin response metrics were calculated and mean values were calculated by day at the trial level. Confidence intervals (90%) were determined by taking the 5th and 95th percentiles of the 100 trial means. Specific populations of interest were obesity (BMI > 30 kg/m^2^) relative to non-obesity (BMI ≤ 30 kg/m^2^) and renal impairment (CrCL < 90 mL/min) relative to normal renal function (CrCL ≥ 90 mL/min).

**Figure 1:**
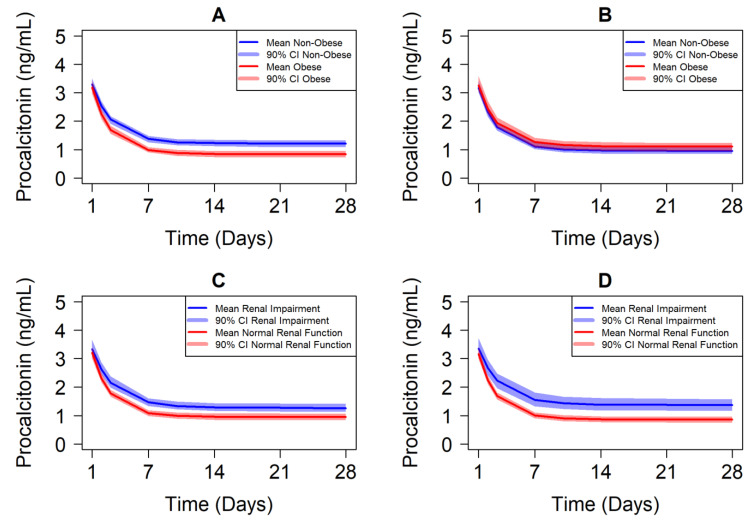
Procalcitonin Response Versus Study Day by Patient-Specific Population Across Simulated Trials. Solid lines depict the mean daily average procalcitonin (PCT) level over time from across the 100 simulated trials with the shaded region corresponding to the 90% confidence interval (CI) of the mean response. Figures are organized by expectation of response to be similar or different across obese and non-obese (**A**,**B**), and renal impairment and normal renal function (**C**,**D**) over time.

**Figure 2:**
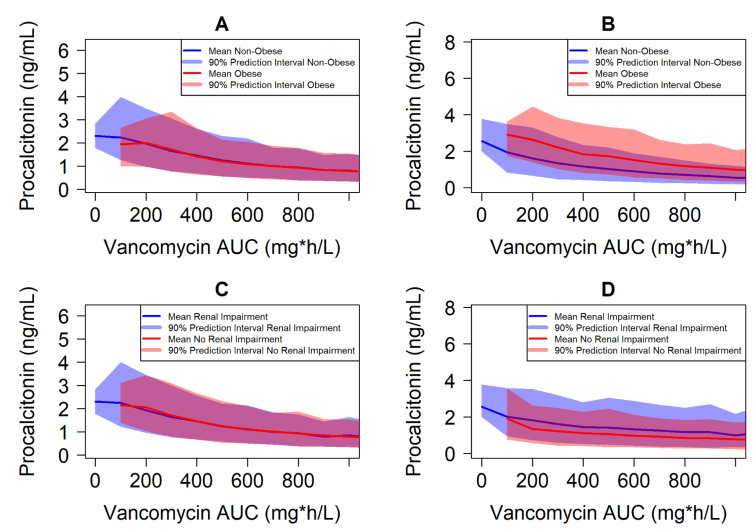
Procalcitonin Response Versus Day 14 Vancomycin AUC by Patient-Specific Population Across All Simulated Patients. Solid lines depict the mean daily average procalcitonin (PCT) level versus individual Day 14 vancomycin area under the curve (AUC) binned in 100 mg*h/L increments across all 125,000 simulated patients with the shaded region corresponding to the 90% prediction interval (PI) of individual response. Figures are organized by expectation of response to be similar or different across obese and non-obese (**A**,**B**), and renal impairment and normal renal function (**C**,**D**) over exposure.

Results are depicted for trial-level response versus time in Figure 1 and individual-level response versus individual Day 14 exposure in Figure 2. Weight-based dosing with the dosing interval determined based on renal function did not lead to exposure-matching across specific populations in the absence of adaptive feedback (TDM). The geometric mean Day 14 vancomycin AUC was 1.7-fold higher in simulated obese patients compared to non-obese patients, as well as 1.6-fold higher in simulated patients with normal renal function compared to those with some degree of renal impairment. This reflects dosing based on total body weight with clearance influenced only by creatinine clearance in the PK model. The specific populations with higher exposure have greater response on average under the assumptions that exposure–response is constant in all populations (Figure 1, left panels). Indeed, individual response with Day 14 AUC overlaps completely for each specific population under this assumption (Figure 2, left panels).

However, it is possible that the exposure–response relationship is not conserved across all sub-populations. When covariate effects for intrinsic patient factors of BMI and CrCL are introduced on procalcitonin response, different trends are visualized. Obese and non-obese patients demonstrate similar trial-level response with time (Figure 1, top right panel) despite 1.7-fold higher exposure in obese patients due to the decreased responsiveness of individual obese patients to vancomycin exposure (Figure 2, top right panel). Conversely, the patients with normal renal function have even greater separation of trial-level response versus time (Figure 1, bottom right panel) due to the combination of 1.6-fold higher exposure and greater individual responsiveness of procalcitonin to vancomycin treatment (Figure 2, bottom right panel) among those with normal renal function. Notably, the 90% prediction interval (PI) covering the 5th to 95th percentiles of individual patient response overlaps significantly for all specific populations, which highlights the importance of adaptive dosing (TDM) to optimize individual outcomes in the setting of narrow therapeutic index antibiotics. These simulations help to illustrate the potential to better identify doses that ensure optimal efficacy outcomes in specific populations where exposure-matching may miss the mark.

While this case study is offered as a potential approach for advancing our approach to dose optimization, it has not been tested and validated. Individual drugs may have the potential to exert anti-inflammatory effects that are independent of their antimicrobial effects. In the above case scenario, we used an indirect response model that did not account for the possibility that the drug (vancomycin in this case) may independently act on the production or elimination of procalcitonin. Deciphering the independent effect of the drug on the kinetics of biomarker production and the elimination process would require additional studies in healthy volunteers challenged with LPS. It is also not known whether biomarker kinetic information gained from one drug can be used to model another drug, and so may require validation for each drug on an independent basis. While this adds scientific complexity, it is an important direction that should be pursued to advance next-generation models of precision dosing in specific populations.

## 6. Summary and Future Directions

The current review highlights the state of optimal empiric antibacterial dose selection that is driven for the most part by exposure-matching in specific populations. The inclusion of antibacterial potency measures such as the minimum inhibitory concentration in order to index these PK measures extends their translatability but does not incorporate differences in host response. Current antibacterial clinical trial designs are also underpowered to gain insights on potential differences in response in subpopulations. Increasing the sample size is not always feasible due to time extension and costs that are a disincentive for drug development. In essence, this discipline needs to advance from “is there a treatment effect?” to “does the drug effect increase with higher doses?” to “what are the characteristics of the exposure-response relationship in subpopulations?” At present, exposure–response analyses are often performed using simple regression methods instead of time-course models. Continuous response measurements and ordered categorical data as a part of clinical endpoints afford the potential for better exposure–response modeling. Daily measurement of an efficacy biomarker, for example, that is coupled with non-linear mixed effects modeling allows for better delineation of the exposure–response relationships. These models also create an opportunity to simulate and guide clinical trial participant enrichment strategies to validate hypotheses. However, it is important to acknowledge that this will add complexity and other modeling challenges, such as: (1) Should participants with missing exposure measurements be included or excluded from the analysis?; (2) Will the imputation of efficacy endpoints bias the results when participants drop out of the study?; (3) Have all relevant confounding covariates been accounted for in the analysis? [54]. As expected, it is not possible to fully validate these assumptions, but sensitivity analyses can be performed to gain confidence in these estimates.

Numerous case examples exist in the literature that have used exposure–response models of efficacy to extrapolate doses in specific populations [55,56,57,58]. In recent work, a longitudinal exposure–response model was used to extend the dosing of a biologic from adults to pediatric patients with psoriasis [59]. This joint modeling framework allowed for the extrapolation of clinical efficacy data in adults to pediatric patients by using an ordered categorical endpoint (Physician’s Global Assessment) and a quantitative rating score (Psoriasis Area and Severity Index) [59]. These modeling approaches can be applied to antibacterial drug development given that similar exposure and response measurements are taken. Continued translational work in this domain is of critical importance to advance model-informed drug development and lower the cost of antimicrobial market entry. Recent work testing bootstrap-based and Bayesian-based methods to estimate the probability of concluding the non-inferiority of the exposure–response relationship of some narrow therapeutic index drugs also offers a useful template for establishing these matching criteria [60]. Building expert consensus on exposure–response modeling and establishing matching criteria across specific populations are needed and worthy of regulatory, academic, and industry attention.

## Figures and Tables

**Table 1:** Efficacy endpoints and potential biomarkers of efficacy by key indication sought for recently approved antibiotics.

Therapeutic Indication	Primary Endpoint	Secondary Endpoint	Potential Continuous and Ordinal Endpoint Measurements
Acute Bacterial Skin and Skin Structure Infections (ABSSSI)	Percent reduction (≥20% typically) in lesion size at 48 to 72 h	Resolution of ABSSSI at 7 to 14 days after therapy completion	Lesion size surface area by serial image analysis Symptom scores (e.g., pain)
Community-Acquired Bacterial Pneumonia	Improvement in at least two symptoms (with no worsening) at day 4 All-cause mortality at 28 days if including severe cases	Improvement in at least two symptoms (with no worsening) at day 4 and vital signs Clinical outcome at end of therapy or at a fixed predefined time point	Change in systemic biomarkers such as C-reactive protein, procalcitonin, calprotectin, presepsin, etc.
Hospital-Acquired Bacterial Pneumonia (HABP) and Ventilator-Associated Bacterial Pneumonia (VABP)	All-cause mortality at any time between 14 and 28 days	(1) Resolution of signs and symptoms of HABP/VABP at approximately 7 to 14 days after the completion of antibacterial drug therapy, (2) days spent in the hospital, and (3) days spent on mechanical ventilation (for VABP and ventilated-HABP patients)	Clinical pulmonary infection scores, procalcitonin values
Nosocomial Pneumonia	All-cause mortality at 14 days	Clinical and microbiological outcomes at Test of Cure or early and later time points	SOFA scores Clinical pulmonary infection score
Complicated Urinary Tract Infections (cUTI)	Microbial Eradication Clinical Cure	Microbial eradication rate Clinical response at the Test of Cure	Reduction in urine bacterial colony forming units

## Data Availability

Not applicable.

## Supplementary Materials

The following are available online at https://www.mdpi.com/article/10.3390/antibiotics10111368/s1, Table S1: Vancomycin Dosing Regimen in Simulated Clinical Trials, Table S2: Summary of Covariate Values in the Source NHANES Datasets, Code S1. Clinical Trial Simulation 1 (No Specific Population Effect)—Template Control Stream, Code S2. Clinical Trial Simulation 1 (No Specific Population Effect)—Template Control Stream
