# Supplementary material for: Translation of Pharmacodynamic Biomarkers of Antibiotic Efficacy in Specific Populations to Optimize Doses
Source: Antibiotics (Basel). 2021 Nov 9;10(11):1368. doi: 10.3390/antibiotics10111368 (PMC8614818; doi:10.3390/antibiotics10111368)
Supplement: Supplementary file 1 [file antibiotics-10-01368-s001.zip › antibiotics-1422316-supplementary.pdf]

antibiotics

MDPI

SUPPLEMENTARY MATERIAL

Review

Translation of Pharmacodynamic Biomarkers of Antibiotic Efficacy in Specific Populations to Optimize Doses

Manjunath P. Pai [1,*] and Ryan L. Crass [2]

[1] Department of Clinical Pharmacy, College of Pharmacy, University of Michigan, Rm 2568, 428 Church St., Ann Arbor, MI 48109, USA
[2] Ann Arbor Pharmacometrics Group, Ann Arbor, MI 48108, USA; Ryan.Crass@a2pg.com
* Correspondence: amitpai@med.umich.edu; Tel.: +1-734-647-0006

Table S1. Vancomycin Dosing Regimen in Simulated Clinical Trials.

Creatinine Clearance	Vancomycin Dose	Dosing Interval
>120 mL/min	15 mg/kg total body weight	Every 8 hours
80-119 mL/min		Every 12 hours
60-79 mL/min		Every 18 hours
40-59 mL/min		Every 24 hours
30-39 mL/min		Every 36 hours
20-29 mL/min		Every 48 hours
< 20 mL/min		Every 72 hours

Table S2. Summary of Covariate Values in the Source NHANES Datasets.

Covariate	Values
**N**	8121
**Sex**	
Male, n(%)	
Female, n(%)	
**Age (years)**	
Mean	49.4
Median (IQR)	50 (34-64)
Min, Max	18, 80
**Weight (kg)**	
Mean	83.4
Median (IQR)	79.8 (67.4-95.5)
Min, Max	32.6, 254.3
**Body Mass Index (kg/m^2)**	
Mean	29.9
Median (IQR)	28.7 (24.7-33.7)
Min, Max	14.2, 92.3
**Creatinine Clearance (mL/min)**	
Mean	118
Median (IQR)	110 (82.9-142)
Min, Max	4.71, 490

Code S1. Clinical Trial Simulation 1 (No Specific Population Effect) – Template Control Stream

```
$PROBLEM VANCOMYCIN ER - CONTINUOUS BIOMARKER
     ;Source PK Model: Yamamoto M et al. J Clin Pharm Ther. 2009;34(4):473-483.
     ;Source PCT Rate Constant: Aulin LBS et al. Clin Pharmacol Ther. 2021;110(2):346-360
     ;Source Baselin PCT: Leli C et al. Dis Markers. 2015;2015:701480.
$INPUT ID TIME AMT DOSE II ADDL DV EVID MDV CMT SEXF AGEYR WTKG HTCM BMI SCR CRCL SEQN

$DATA sim.csv
      IGNORE=@

$SUBROUTINE ADVAN6 TRANS1 TOL=6

$MODEL
COMP = (CENTRAL)
COMP = (PERIPH)
COMP = (BIOMRK)

$PK

;;PK MODEL PARAMETERS;;
     IF(CRCL.LT.85) THEN
      TVCL = THETA(1) + THETA(2)*CRCL
     ELSE
      TVCL = THETA(3)
     ENDIF

     TVV1 = THETA(4)*WTKG
     TVQ = THETA(5)
     TVV2 = THETA(6)

     CL = TVCL*EXP(ETA(1))
     V1 = TVV1*EXP(ETA(2))
     Q = TVQ*EXP(ETA(3))
     V2 = TVV2*EXP(ETA(4))

     K12  = Q/V1
     K21  = Q/V2
     K10  = CL/V1

     S1   = V1                ; dose = mg, conc = mcg/mL = mg/L
;;PK MODEL PARAMETERS;;

;;ER MODEL PARAMETERS;;
     BASE = THETA(8)*EXP(ETA(5))
     KOUT = THETA(9)*EXP(ETA(6))
     IMAX = THETA(10)
     IC50 = THETA(11)*EXP(ETA(6))

     A_0(3) = BASE
     KIN = BASE*KOUT
;;ER MODEL PARAMETERS;;


$DES

;;ER MODEL;;
C1 = A(1)/S1
IDRUG = (IMAX*C1)/(C1+IC50)
;;ER MODEL;;

DADT(1) = K21*A(2)-(K12 + K10)*A(1)
DADT(2) = K12*A(1)-K21*A(2)
DADT(3) = KIN*(1-IDRUG) - KOUT*A(3)

$ERROR (OBSERVATION ONLY)
CP = A(1)/S1
```

```
IF(CMT.EQ.1) THEN
W = SQRT(THETA(7)**2*F**2)
IPRED=F
ENDIF

IF(CMT.EQ.3) THEN
W = THETA(12)
IPRED=LOG(F)
ENDIF

IF(F.GT.0) THEN
Y=IPRED+W*EPS(1)
ELSE
Y=0
ENDIF


$THETA
0.32 FIX      ;1 CL AT CRCL=0 [L/h]
0.0322 FIX         ;2 CL SLOPE WITH CRCL < 85 [L/h/(mL/min)]
3.83 FIX      ;3 CL CRCL >/= 85 [L/h]
0.478 FIX     ;4 V1 PER WTKG IN PATIENTS [L/kg]
8.81 FIX      ;5 Q [L/h]
60.6 FIX      ;6 V2 IN PATIENTS [L]
0.143 FIX     ;7 PROP RE

3.6 FIX       ;8 BASELINE PCT [ng/mL]
0.0289 FIX         ;9 KOUT PCT [1/h]
1 FIX              ;10 IMAX
10 FIX             ;11 IC50 [mg/L]
0.3 FIX       ;11 LOG ADD RE

$OMEGA
0.141 FIX     ;11 ETA1-CL
0.0333 FIX         ;22 ETA2-V1
0.0367 FIX         ;33 ETA3-Q
0.530 FIX     ;44 ETA4-V2

0.105 FIX     ;55 ETA5-BASELINE PCT
0.224 FIX     ;66 ETA6-IC50

$SIGMA
1.000 FIXED

$SIM (09151972) ONLYSIM SUBPROBLEM=1

$TABLE    ID TIME AMT DOSE II ADDL EVID MDV CMT SEXF AGEYR WTKG HTCM BMI SCR CRCL SEQN
          PRED IPRED DV
          NOPRINT NOAPPEND ONEHEADER FILE=sim.tab
```

Code S2. Clinical Trial Simulation 1 (No Specific Population Effect) – Template Control Stream

```
$PROBLEM VANCOMYCIN ER - CONTINUOUS BIOMARKER
     ;Source PK Model: Yamamoto M et al. J Clin Pharm Ther. 2009;34(4):473-483.
     ;Source PCT Rate Constant: Aulin LBS et al. Clin Pharmacol Ther. 2021;110(2):346-360
     ;Source Baselin PCT: Leli C et al. Dis Markers. 2015;2015:701480.
$INPUT ID TIME AMT DOSE II ADDL DV EVID MDV CMT SEXF AGEYR WTKG HTCM BMI SCR CRCL SEQN

$DATA sim.csv
      IGNORE=@

$SUBROUTINE ADVAN6 TRANS1 TOL=6

$MODEL
COMP = (CENTRAL)
COMP = (PERIPH)
COMP = (BIOMRK)

$PK

;;PK MODEL PARAMETERS;;
     IF(CRCL.LT.85) THEN
      TVCL = THETA(1) + THETA(2)*CRCL
     ELSE
      TVCL = THETA(3)
     ENDIF

     TVV1 = THETA(4)*WTKG
     TVQ = THETA(5)
     TVV2 = THETA(6)

     CL = TVCL*EXP(ETA(1))
     V1 = TVV1*EXP(ETA(2))
     Q = TVQ*EXP(ETA(3))
     V2 = TVV2*EXP(ETA(4))

     K12  = Q/V1
     K21  = Q/V2
     K10  = CL/V1

     S1   = V1                ; dose = mg, conc = mcg/mL = mg/L
;;PK MODEL PARAMETERS;;

;;ER MODEL PARAMETERS;;
     TVBASE = THETA(8)
     TVKOUT = THETA(9)
     TVIMAX = THETA(10)
     TVIC50 = THETA(11)*((CRCL/110)**THETA(13))*((BMI/30)**THETA(14))

     BASE = TVBASE*EXP(ETA(5))
     KOUT = TVKOUT*EXP(ETA(6))
     IMAX = TVIMAX
     IC50 = TVIC50*EXP(ETA(6))

     A_0(3) = BASE
     KIN = BASE*KOUT
;;ER MODEL PARAMETERS;;


$DES

;;ER MODEL;;
C1 = A(1)/S1
IDRUG = (IMAX*C1)/(C1+IC50)
;;ER MODEL;;

DADT(1) = K21*A(2)-(K12 + K10)*A(1)
DADT(2) = K12*A(1)-K21*A(2)
DADT(3) = KIN*(1-IDRUG) - KOUT*A(3)
```

```
$ERROR (OBSERVATION ONLY)
CP = A(1)/S1

IF(CMT.EQ.1) THEN
W = SQRT(THETA(7)**2*F**2)
IPRED=F
ENDIF

IF(CMT.EQ.3) THEN
W = THETA(12)
IPRED=LOG(F)
ENDIF

IF(F.GT.0) THEN
Y=IPRED+W*EPS(1)
ELSE
Y=0
ENDIF


$THETA
0.32 FIX      ;1 CL AT CRCL=0 [L/h]
0.0322 FIX        ;2 CL SLOPE WITH CRCL < 85 [L/h/(mL/min)]
3.83 FIX      ;3 CL CRCL >/= 85 [L/h]
0.478 FIX     ;4 V1 PER WTKG IN PATIENTS [L/kg]
8.81 FIX      ;5 Q [L/h]
60.6 FIX      ;6 V2 IN PATIENTS [L]
0.143 FIX     ;7 PROP RE

3.6 FIX       ;8 BASELINE PCT [ng/mL]
0.0289 FIX        ;9 KOUT PCT [1/h]
1 FIX             ;10 IMAX
10 FIX            ;11 IC50 [mg/L]
0.3 FIX       ;12 LOG ADD RE
-1 FIX            ;13 CRCL on IC50 [REF: 60 mL/min]
3 FIX             ;14 BMI on IC50 [REF: 30 kg/m^2]

$OMEGA
0.141 FIX     ;11 ETA1-CL
0.0333 FIX        ;22 ETA2-V1
0.0367 FIX        ;33 ETA3-Q
0.530 FIX     ;44 ETA4-V2

0.105 FIX     ;55 ETA5-BASELINE PCT
0.224 FIX     ;66 ETA6-IC50

$SIGMA
1.000 FIXED

$SIM (09151972) ONLYSIM SUBPROBLEM=1

$TABLE    ID TIME AMT DOSE II ADDL EVID MDV CMT SEXF AGEYR WTKG HTCM BMI SCR CRCL SEQN
          PRED IPRED DV
          NOPRINT NOAPPEND ONEHEADER FILE=sim.tab
```
